# Engaging young African American women breast cancer survivors: A novel storytelling approach to identify patient‐centred research priorities

**DOI:** 10.1111/hex.13021

**Published:** 2020-01-09

**Authors:** Alice Yan, Sandra Millon-Underwood, Alonzo Walker, Caitlin Patten, Debra Nevels, Keith Dookeran, Rose Hennessy, Mary Jo Knobloch, Leonard Egede, Melinda Stolley

**Affiliations:** ^1^ Joseph J. Zilber School of Public Health University of Wisconsin Milwaukee Milwaukee WI USA; ^2^ College of Nursing University of Wisconsin Milwaukee Milwaukee WI USA; ^3^ Division of Surgical Oncology Breast Care Center Froedtert & The Medical College of Wisconsin Milwaukee WI USA; ^4^ American Cancer Society – North Region Waukesha WI USA; ^5^ School of Medicine and Public Health University of Wisconsin Madison Madison WI USA; ^6^ Division of General Internal Medicine Medical College of Wisconsin Milwaukee WI USA; ^7^ Division of Hematology and Oncology Medical College of Wisconsin Milwaukee WI USA

**Keywords:** African American, patient engagement, patient‐centred approach, storytelling, young breast cancer survivors

## Abstract

**Background:**

Patient‐centredness is considered an essential aspiration of a high‐quality health‐care system, and patient engagement is a critical precursor to patient‐centred care.

**Objectives:**

To engage patients, health‐care providers and stakeholders in identifying recommendations to address research and practice gaps that impact young African American breast cancer survivors.

**Methods:**

This paper reported an approach for research priority setting. This approach applies an engagement process (January‐September 2018) of using patient and stakeholder groups, patient storytelling workshops and a culminating storytelling conference in Wisconsin to generate relevant research topics and recommendations. Topics were prioritized using an iterative engagement process. Research priorities and recommendation were ranked over the conference by counting participants’ anonymous votes.

**Results:**

One hundred attendees (43 patients/family members, 20 providers/researchers and 37 community members) participated in the conference. Five topics were identified as priorities. The results showed that three priority areas received the most votes, specifically community outreach and education, providing affordable health care and engaging in complementary care practice. Stakeholders also agreed it is critical to ‘include youth in the conversation’ when planning for cancer support and educational programmes for caregivers, friends and family members.

**Conclusion:**

Storytelling as a patient engagement approach can build trust in the patient‐research partnership, ensure that patients are meaningfully engaged throughout the process and capture the diversity of patient experiences and perspectives.

## 1 INTRODUCTION

Breast cancer is the most common cancer diagnosed in women and the second leading cause of cancer‐related death in US women. Most breast cancers are diagnosed in post‐menopausal women, but approximately 11% of all new breast cancer cases in the United States are found in women younger than 45 years of age.^1^ Young women with breast cancer may face unique challenges related to relationships, parenting, finances and employment compared to their older counterparts. Evidence suggests that young African American female breast cancer survivors (YAABCS) face even greater challenges. The breast cancer mortality rates are three times higher in young African American women than in young Caucasian women.^1^ Additionally, YAABC survivors are more often diagnosed with biologically more aggressive disease (ie triple negative) and/or metastatic disease leading to poorer prognosis, aggressive treatments, long‐term treatment‐related side‐effects and unique psychosocial concerns. In 2018, the New American College of Radiology (ACR) and Society of Breast Imaging (SBI) published breast cancer screening guidelines that were the first to recognize that African American women are at high risk of developing breast cancer and need further consideration for earlier (ie screening to begin prior to age 40) and/or more intensive screening.^2^ In addition, it is well documented that Black women are more likely to experience delays in follow‐up to abnormal mammography and treatment initiation,^3^, ^4^, ^5^, ^6^ which may be particularly salient for YAABCS. Despite these disparities, few efforts have sought to bring YAABCS, health‐care providers and researchers together to inform research priorities that will lead to improved breast cancer outcomes and care.

Patient engagement is an established strategy that can be used to inform research to address health disparities and improve the delivery of effective and responsive health‐care services.^7^, ^8^, ^9^ Regardless, there is limited consensus on how best to engage patients.^7^

The overarching goal of the current study was to bring together YAABCS, health‐care providers and stakeholders to identify recommendations to address research and practice gaps that impact YAABCS.^10^ This study contributes to the literature on patient engagement in several ways. First, it contributes to the literature on providing a detailed process to address research priority setting for YAABCS, as the literature suggests that priority setting between researchers and stakeholders may improve research relevance and value.^11^, ^12^ Second, we use an innovative patient‐centred storytelling approach to effectively engage YAABC survivors. Storytelling is deeply rooted in African American culture.^13^ Storytelling in the patient's own voice has the power to directly and more effectively confront a patient's health concerns, outcomes of interests, as well as barriers to receiving care across the cancer control continuum.^10^, ^14^ This approach was used in Henry Ford Health System (Detroit, Michigan, USA), which successfully led to the development of a set of patient‐centred comparative effectiveness research questions.^15^ Third, we apply key patient/stakeholder engagement principles and best practices from the Patient‐Centered Outcomes Research Institute (PCORI).^16^

Herein, we present the collaborative learning process and findings from the African American young breast cancer survivor storytelling project that culminated in a storytelling conference. The aims of this project were to: (a) learn the health concerns, outcomes of interests, as well as barriers to receiving care across the cancer control continuum from YAABC survivors through storytelling; (b) generate a list of research questions as well as practice and policy recommendations to address cancer disparities among young African American women; and (c) facilitate new relationships among patient, researchers, clinicians and policymakers.

## 2 METHODS

### 2.1 Study design

This study reported an approach for research priority setting. This approach we outlined in the paper applies an engagement process of using patient and stakeholder groups, patient storytelling training workshops and a culminating storytelling conference to generate relevant research topics and questions to improve breast cancer care in YAABCS.

### 2.2 Recruitment

We established two groups: (a) a patient advisory group of 10 female YAABC survivors; and (b) a stakeholder group comprised of six members, including health‐care providers, academic researchers and representatives from patient advocate groups currently engaged in topics related to breast cancer and/or breast cancer disparities. Patients (YAABC) were eligible if they were African American women living in Milwaukee County with a history of breast cancer diagnosed between the ages of 20 and 45 years old. We used the age of 45 as the cut‐off to define young breast cancer patients based on the CDC’s definition.^1^ We focused on Milwaukee County based on data showing that nearly 90% of African Americans in Wisconsin live in Milwaukee County. Furthermore, breast cancer deaths in Wisconsin are highest in Milwaukee County.^17^ Within Milwaukee County, African American women have the highest age‐adjusted breast cancer mortality rate (34.3 per 100 000), 1.7 times that of white women (20.3 per 100 000)^17^ and far exceeding the Healthy People 2020 target of 20.7 or less.^18^

Three approaches were used to recruit participants and conference attendees: (a) physicians and nurses recruited patients during their clinical visits (patients only); (b) existing community‐based partnerships were leveraged to recruit through breast cancer support groups, local African American beauty salons, churches, neighbourhood health centres and social service agencies; and (c) advertisements and programming on V100.7, a popular radio station in the African American community, provided information on the opportunity to join the patient or stakeholder group and/or attend the storytelling conference. The study was exempt from the Institutional Review Board as it only involves the engagement activities related to a conference.

### 2.3 Stakeholder group meetings

Between March and May 2018, our research team convened two key discussion groups: one with the patient advisory group and one with the other stakeholders to generate and refine topics to be discussed in the upcoming storytelling conference. For each group, we asked ‘what are the most relevant and important questions/issues across the cancer control continuum (from prevention to survivorship) that you believe would improve breast cancer outcomes among young African American women’. We also solicited patient‐related concerns and challenges (eg screening, diagnosis, treatment and survivorship) among the patient advisory group. The topics from these groups were summarized by themes, which were then voted on at the beginning of the storytelling conference for further discussion among conference attendees.

Patient advisory group members received $200 for their time and travel, and stakeholder group members received $100. Patients were compensated more based on their greater time commitment. Our reimbursement practice adhered to PCORI’s best practice on ‘compensation and reimbursement of out‐of‐pocket expenses’.^16^

### 2.4 Storytelling workshops

Prior to the storytelling conference (May‐August 2018), we conducted a series of six storytelling workshops. The purposes of the workshop were to identify the top five concerns and challenges of African American breast cancer survivors and to help survivors identify, structure, practice and share their personal stories effectively. To make the storytelling process more concise and captivating for the audience, we partnered with a local non‐profit storytelling organization, Ex Fabula, and hired an African American storytelling coach to facilitate the workshops. The coach managed six 120‐minute conversational style workshops for the 10 participants, during which participants were taught the basics of storytelling and ways to be successful in the art of storytelling. Workshop topics included identifying their challenges and concerns, creating story ideas (to illustrate these challenges and concerns) and story structure, determining conference agenda (ie which stories, what order, presentation time) and others. Upon completion of the workshops, participants were invited to record their stories. These stories, which highlight the need to develop evidence‐based interventions and policies, were posted on YouTube with participant consent. https://www.youtube.com/watch?v=GjNHRCmydmM

### 2.5 Conference overview

The storytelling conference titled, ‘Sisters We Thrive, Stories We Tell’, was scheduled on a Saturday from 8:30 am to 2 pm in Milwaukee, Wisconsin. YAABC survivors, their family members or caregivers, health researchers, clinicians, representatives from patient advocate groups and other community stakeholders were invited. The conference included keynote speakers, storytelling and discussion groups. To inform discussion group topics, attendees received a short questionnaire upon arrival that requested information on their identified gender and race/ethnicity. The conference attendees were also asked to rank by order of importance of the eight research topics/themes generated by the stakeholder meetings and storytelling workshops (Table 2). Then, conference staff summarized the ranking. Of the eight topics related to improved care and outcomes for YAABCS, five were ranked as most important (Table 2). The conference kicked off with a general session of keynote presentations by three local health disparity researchers, followed by a two‐hour StorySlam (ie storytelling event)^19^ coordinated by the storytelling coach. The ten participants who participated in the storytelling workshops shared their stories with the conference attendees. A variety of formats was used including: five longer stories, short anecdotes and UltraShorts.^20^, ^21^ Other conference attendees had the chance to participate actively at the event, if they desired, by writing a few sentences on a piece of paper based on reflections and attendee stories and having them read on stage as UltraShorts.

Following the storytelling, attendees were encouraged to join one of five discussion groups targeting a particular topic that was voted as high priority at the beginning of the conference. Each group had a facilitator who followed a five‐step protocol (Table 1) to lead an interactive brainstorming session on solutions related to the group topic. At the end of the round table discussion, the facilitator asked participants at each table to vote on their top 3 statements or solutions they believed would have the most impact.

**Table 1:** Flow diagram showing procedures of stakeholder engagement and the storytelling conference

Procedures	Stakeholders	Programme inputs/activities	Outcomes
Establish patient stakeholder group	Ten young African American breast cancer survivors	Conducted a key discussion group to answer the question ‘what are the most relevant and important questions/issues that you *believe* can improve breast cancer outcomes among young African American women’	Summarize findings
Establish other stakeholder group	Six members including providers, researchers, health‐care system representative and patient advocate group	Conducted a key discussion group to answer the question ‘what are the most relevant and important questions/issues that you *believe* can improve breast cancer outcomes among young African American women’	Summarize findings
Storytelling Workshops	Ten young African American breast cancer survivors	A storytelling coach facilitated six 120‐min workshops to (a) identify top five concerns and challenges of African American breast cancer survivors; (b) teach YAABC survivors how to communicate their stories effectively As the deliverable of the workshops, patient stakeholders will develop a short personal story that is most important to them around concerns and challenges relevant to cancer care	Patients‐centred stories were developed by survivors
Patient stories in a film	Five young African American breast cancer survivors	Developed a documentary film of patient stories developed via workshops to raise awareness of important public health issues among the wide public	Patients‐centred stories were featured in a documentary film
Research team reviewed findings from two discussion groups and patient stories and identified categories that are most relevant to YAABC to be further discussed at the storytelling conference			
Interactive storytelling conference	Attendees include patient/family member (n = 43), researchers/health professionals (n = 20) and community members (n = 37)		
Step 1. Initial voting		At registration, participants were asked to select only one topic that they believe is most important to address racial disparities in breast cancer mortality (death rates) and poor 5‐y survival rate among African American young breast cancer patients/survivors in Southeast Wisconsin	
YAABC survivor storytelling (Story Slam)			
Round table discussion			
Step 2. Determine topics for round table discussion		Based on selections of participants from step 1, we determined the top five topics (seven tables) of interest. One facilitator is assigned to each table (round table discussion)	At the end of round table discussion, participants will have participated in an in‐depth conversation of possible solution/recommendation to address racial disparities in breast cancer mortality and poor five‐year survival rate among YAABC survivors when compared to young White women
Step 3. Discuss and rank the solutions		Attendees were encouraged to join one of the tables that have the topic interests them the most. At each table, a facilitator leads the discussion and asks round table participants to list what are the possible solutions (how to fix the problem) related to that topic. At the end of the round table discussion, the facilitator asked participants to rank the top 3 solutions by raising their hands. Each person can only raise their hand up to three times	
Step 4. Problem‐solving statements		Programme staff gathered together the top 3 solutions from each table. Each solution was written in a statement. We had one table with six statements (rather than 3). Then, programme staff transferred those statements into four self‐adhesive whiteboard papers	
Step 5. Participants’ final ranking of recommendations or solution statements		MC read out lead the list of all recommendation or solution statements on the whiteboard papers. MC asked participants to select the top three statements they believe are most important to address the problem of having higher death rates and poor survival rate among African American young breast cancer survivors in Southeast Wisconsin	

The final voting process started with a master of ceremonies (MC) reading out the list of all recommendation/solution statements on the whiteboard. The MC asked all attendees (each one was given a unique sticky colour dot that distinguished their role as either patient/family member, researchers/health professionals or community members) to use the dot to vote for the top 3 statements they believed were most important in addressing the problem of having higher death rates and poorer survival rates among YAABCS in Milwaukee. This approach aligns with PCORI’s standard process for prioritizing patient‐centred research questions, which asks stakeholders to judge topics based in part on the topic's importance to the individual themselves.^22^

## 3 RESULTS

### 3.1 Prioritized research agenda findings

#### 3.1.1 Patient advisory and stakeholder group: generating research topics

The stakeholder meetings (with the patient advisory group and the stakeholder group) and the patient‐centred storytelling workshops generated a list of eight research topics most relevant to YAABC survivors. Those topics (shown in Table 2) were used as the starting point for further discussions in the storytelling conference.

**Table 2:** Initial voting on a list of eight topics: select one topic that you believe is most important to help African American young women have an enhanced breast cancer screening, obtain timely care about an abnormal mammogram, recover from treatments and live longer with a good quality of life in Southeast Wisconsin

Topics	Frequency	Percent (ranking)
Addressing access to care and insurance coverage, such as eligibility for early detection (based on age), access to quality mammography, referrals to genetic counseling	8	**21.6 (#1)**
Providing support and educational programs for caregivers, friends, and family members of breast cancer survivors	7	**18.9 (#2)**
Managing breast cancer treatment side effects (tiredness, headaches, pain and numbness, lymphedema, bone loss and osteoporosis, heart problems, menopause, sexual difficulties, infertility, chemo brain), and addressing their impact on quality of life and minimizing the impact of financial hardship experienced by cancer survivors	6	**16.2 (#3)**
Enhancing patient understanding of treatment options (standard of care, clinical trials) and right to seek second opinions	5	**13.5 (#4)**
Incorporating spirituality and positive thinking during and after treatment	4	**10.8 (#5)**
Learn how to better cope with changes in personal life during and after treatment (relationships, the possibility of early menopause caused by chemotherapy, fertility, sexuality, psychological distress, disruption of employment, others)	3	8.1
Encourage individuals to have a positive lifestyle change (be more active, eating a healthy diet, follow up screening, quit smoking, and connect or reconnect with your primary care provider) to establish a long‐term relationship to check for other health problems such as diabetes, high blood pressure, heart disease, bone loss, and other health conditions	3	8.1
Developing a culturally relevant survivorship care plan. A survivorship care plan is a written or electronic document filled out by your oncologist at the end of treatment that helps you and your medical team coordinate your future care. It should list all therapies you received as well as other medical information relevant to your diagnosis and ongoing monitoring and treatment	1	2.7
Total	37	100.0

### 3.2 Storytelling conference

#### 3.2.1 Storytelling conference: participants

Of the 100 participants attending the conference, 37 (37%) completed the self‐administered questionnaire: the majority were women (89.2%, n = 33) and African American (84%, n = 31).

#### 3.2.2 Storytelling conference: ranking topics

Of the eight topics related to improved care and outcomes for YAABCS that were generated by the stakeholder meetings and storytelling workshops, conference attendees selected five. The most highly ranked topics were (Table 2):

1. Addressing access to care and insurance coverage, such as eligibility for early detection (based on age), access to quality mammography and referrals to genetic counselling.
2. Providing support and educational programmes for caregivers, friends and family members of breast cancer survivors.
3. Managing breast cancer treatment side‐effects (tiredness, headaches, pain and numbness, lymphedema, bone loss and osteoporosis, heart problems, menopause, sexual difficulties, infertility and chemo brain), addressing their impact on quality of life and minimizing the impact of financial hardship experienced by cancer survivors.
4. Enhancing patient understanding of treatment options (standard of care and clinical trials) and right to seek second opinions.
5. Incorporating spirituality and positive thinking during and after treatment.

#### 3.2.3 Storytelling conference: generating recommendations or solutions

During the round table discussions, attendees (patient/family member [n = 43], researchers/health professionals [n = 20] and community members [n = 37]) generated several recommendations or solution statements to each topic and voted on their top 3. Note that while we expected only 15 (five groups, three recommendations/solutions), we made an exception for one round table whose participants generated six priorities. Priorities from the five groups reflected patient‐, provider‐ and health system‐level factors (see Table 3).

**Table 3:** Solution statements identified as priorities as the result of the final voting

#Priority category 1. Addressing access to care and insurance coverage, such as eligibility for early detection (based on age), access to quality mammography, referrals to genetic counseling	
B1	Health care need to be affordable (co‐pays, sup. Medical supplies, people who don't fall under the poverty line but still can't afford medical expenses)
B2	Information needs to be used (passed on to elected officials, moved past research and into practice)
B3	Resource awareness (communicating, put resources in the hands of the people who need them)
#Priority category 2. Providing support and educational programs for caregivers, friends, and family members of breast cancer survivors	
A1	Community‐driven programs, outreach, and activities
A2	Utilize churches
A3	Include youth in the conversation (schools, other neighborhood locations, talking to friends, creating resources, art/music/IT)
#Priority category 3. Managing breast cancer treatment side effects (tiredness, headaches, pain and numbness, lymphedema, bone loss and osteoporosis, heart problems, menopause, sexual difficulties, infertility, chemo brain), and addressing their impact on quality of life and minimizing the impact of financial hardship experienced by cancer survivors	
D1	How do we build community resources that support healthy living?
D2	How do we build community spaces for fostering communication about side effects?
D3	How do we change the narrative about breast cancer and side effects (such as depression) so that survivors, supporters, and community are comfortable talking about these subjects?
D4	Why do some have side effects and others do not? (Are there links of diet or genetics factors to side effects?)
D5	How come ‘traditional’ providers don't address complementary treatments for side effects?
D6	How do you ensure consistent providers who know you as a patient and not just from your chart?
#Priority category 4. Enhancing patient understanding of treatment options (standard of care, clinical trials) and right to seek second opinions	
C1	Communication is the key. Patients are shut down during clinical visit. Providers should learn how to listen to questions from patients and answer questions
C2	We need more personalized decision‐making tools to help you make decisions that are relevant to you, not other people
C3	If you (patients) don't ask (doctors or other health professionals) questions, the answer is no
#Priority category 5. Incorporate spirituality and positive thinking during and after treatment	
E1	Create a platform to engage in complementary care practices.
E2	Doctors and other health professionals be more open to spirituality and positive thinking
E3	Learn about clinics and other treatment facilities to (learn from and) implement this level of care

#### 3.2.4 Final voting on recommendations and solutions: Comparing and contrasting priorities among different stakeholders (see Table 3; Figure 1)

**Figure 1:**
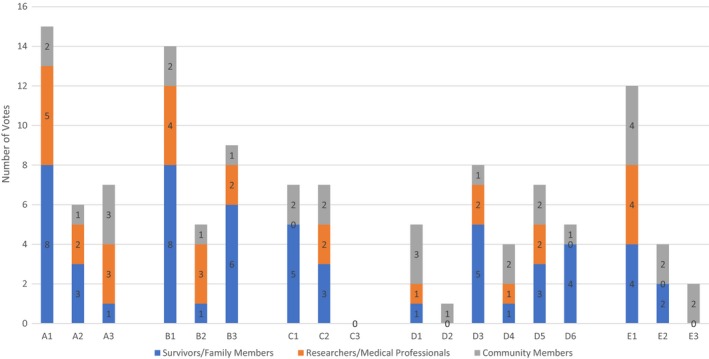
Number of final voting for recommendation or solution statements (by three different types of stakeholders)

The colour dot voting provided a unique opportunity for different stakeholders (patients/family members, researchers/health professionals and community members) to decide what matters most when addressing health disparities among YAABCS. Figure 1 shows the numbers of vote for each statement by three different types of stakeholders. Table 3 lists prioritized recommendation or solution statements as the result of the final voting. The orders of priority statements were determined by the total number of voting ‘dots’. Overall, statements A1 (community outreach and education, vote counts = 15), B1 (affordable health care, vote counts = 14) and E1 (engage in complementary care practice, vote counts = 12) received the most votes.

All voting stakeholders equally agree on the need to ‘create a platform to engage in complementary care practice’ (E1) and use of complementary treatments for side‐effects (D5) as well as using ‘personalized decision‐making tool (C2)’ for patients. Stakeholders also seemed to agree that it is critical to ‘include youth in the conversation’ when planning for cancer support and educational programmes for caregivers, friends and family members (A3).

In contrast, stakeholders had different perspectives on the importance of health insurance (B1) and community outreach (A1, A2, B3), as well as changing the narrative about breast cancer and its side‐effects (D3) in the community. For example, compared to researchers/health professionals, more patients/family or community recognized the importance of providing access to affordable health care (B1), utilizing churches (A2) for community‐based outreach programmes (A1) to help patients in navigating community/health resources (B3), and changing the narrative about cancer and side‐effects (ie depression, so that survivors, supporters and community are comfortable talking about it) (D3).

Surprisingly, three patient‐provider communication‐related statements (C1, D6, E2) were not voted as top priorities by researchers/medical professional (ie received zero voting). They are (a) ‘communication is the key. Patients are shut down during clinical visit. Providers should learn how to listen to questions from patients and answer questions’; (b) ‘how do you ensure consistent providers who know you as a patient and not just from your chart?’; and (c) ‘doctors and other health professionals be more open to spirituality and positive thinking’. Not surprisingly, when compared to patients/family or community members, more researchers/medical professional believed moving research into policy and practice is a priority (B2).

## 4 DISCUSSION

Patient‐centredness is considered an essential aspiration of a high‐quality health‐care system,^23^ and patient engagement is a critical precursor to patient‐centred care.^7^, ^24^ As concluded by the 2014 Institute of Medicine: ‘Prepared, engaged patients are a fundamental precursor to high‐quality care, lower costs and better health’.^25^ In this study, we provide an example of how to actively engage patients and other stakeholders using established best practices, along with a novel storytelling approach.^16^ The engagement approaches including storytelling contribute to the literature on using innovative methods to address the knowledge‐to‐action gap^26^ between the ‘what’ and ‘how’ of patient engagement.^7^ Patient engagement can happen at several levels from inform, in which little or no active participation occurs (eg receiving information via a website or booklet), to empower, in which the goal is to provide patients the necessary tools to feel comfortable with decision‐making power.^27^ Several measures were put in place to ensure genuine and meaningful engagement of patient stakeholders. First, patients were closely involved throughout the research topic generation and prioritization process. Trust was built early between stakeholders and research team through regular dialogue. A fun and interactive storytelling approach empowered patients to use their own voice to directly and more effectively confront their health concerns, outcomes of interests, as well as barriers to receiving care across the cancer control continuum.^10^, ^14^ Second, patient stakeholders had the decision‐making power. A shared and collaborative decision‐making process was used throughout the entire project period to identify research priorities.^16^ We applied a multiple iteration approach that involved face‐to‐face meetings, workshops and a conference round table to prioritize the research topics. These processes adhered to PCORI’s patient engagement principles and allowed stakeholders to judge (ie voting) topics based in part on the topic's importance to the individual themselves.^22^

Consistent with the literature, the patient‐centred research agenda and recommendations in regard to breast cancer outcome disparities are related to patient‐, provider‐ and health system‐level factors.^28^, ^29^ Our patients’ personal stories indicated that lack of insurance, fear of mammogram and treatment delay due to fear of death or lack of insurance all contribute to disparities at the patient level. At the provider level, our patients’ stories showed that some doctors may ignore or inadequately address minority women's serious side‐effects of treatment. Our finding also highlighted the importance of addressing patient‐provider communication factors^29^, ^30^ such as training providers as good listeners and addressing patients’ spirituality needs when necessary.^31^

Health system factors such as higher copayment requirements, lack of a routine source of care, distance to care, fragmentation of care and uneven distribution of screening and treatment resources also exacerbate disparities.^28^, ^32^ Our study findings suggest that pinpointing policy/health system‐level factors that contribute to the root causes of persistent disparities in breast cancer outcomes can help policymakers focus efforts to equalize health‐care access and quality across diverse user populations. Diagnosing breast cancer in younger women (under 40 years old) is more difficult because their breast tissue is generally denser than the breast tissue of older women. On 28 March 2019, the FDA announced important new steps to modernize breast cancer screening and requires states to provide breast density reporting to both patients and referring health providers. Currently, only five states (IL, NY, VT, NJ and CT)^33^ require both density notification and insurance coverage for supplemental imaging for women with dense breast tissue. It is not surprising that a recent JAMA study^34^ stated that ‘Cancer injustice is not a science problem, a technology problem, or a genetics problem. It is a policy problem—simply requires action that equalizes access to and utilization of high‐quality care’.

Our findings also highlight the need to use integrative health care that brings conventional and complementary approaches together for relief of symptoms in cancer patients and survivors. Integrative health care emphasizes a holistic, patient‐focused approach to health care—often including physical, emotional, spiritual, social and community aspects. In North America, 48%‐80% breast cancer survivors use complementary and integrative therapies such as acupuncture, yoga, massage and meditation following diagnosis.^35^ Survivors used these complementary therapies to help manage symptoms and side‐effects when receiving conventional cancer treatment. Although some studies show promising results of using integrative approaches for cancer patients’ and survivors’ symptom management,^35^, ^36^ this line of research is still in its early stages. To our knowledge, no studies have examined younger minority breast cancer survivors’ use of integrative medicine. More robust scientific data about the efficacy and cost‐effectiveness in integrative oncology for managing cancer symptoms as well as addressing spiritual, social and community aspects of patients are needed.

Our unique findings indicated that patients and researchers/doctors may have very different perspectives when it comes to illness. For health professionals, their lens was usually narrowly focused on the disease itself and treatments. For patients, their wide‐angle lens took in the whole of their lives, of which disease was one small part. The challenge ahead for patient‐centred care is helping providers understand that other social determinants^37^ of patients’ life may play a bigger role in leading up to the current situation (ie chronic diseases, medical non‐adherence, higher mortality rates). Only by overcoming this challenge will patients and providers each be able to adjust the angles of their respective lenses so that their vision can come into common focus.

This study has some limitations. First, the patient stakeholders represent highly motivated minority patients. Second, although various methods were used to recruit patient stakeholders, we believe the nature of the project ‘be willing to share your personal stories’ may have limited some hard‐to‐reach YAABC survivors from participating. Therefore, the list of priority research topics may not be generalizable to all young female African American breast cancer patients. Third, our efforts to engage conference attendees to narrow this list to five topics upon arrival captured only a small subset. However, the final voting process did include the voices of nearly all attendees. Additionally, conference participant composition was not evenly distributed among all stakeholder groups. We cannot perform formal statistical analyses on the voting outcomes beyond reporting patterns. Given the objective of the study was to generate patient‐centred research topics and priorities and conference attendees include more patients (survivors), family members or caregivers, and community stakeholders than the researchers or health‐care providers, we believe the priority statements were patient‐centred and community‐driven. Finally, we created separate groups of patients and providers/other stakeholders for identifying research priorities and brought them together at the conference. This approach, although similar to other studies,^9^, ^38^ may not be ideal for increasing interactions between researchers, clinicians, and patients.

Findings have the potential to inform research, practice and policy in addressing health disparities among African American young breast cancer survivors. We anticipated that the next steps are twofold. First, the statewide policymakers, practitioners and decision‐makers within local health‐care systems need to be informed about our findings. The research team has already started the dissemination process. For example, our findings as well as the patient storytelling film were featured and discussed at the statewide cancer summit and health‐care delivery planning meetings. Second, final priorities will be disseminated with researchers so they can utilize this information to guide the design of future research projects. This conference established partnerships between patients, providers and researchers. Together, we plan to investigate many of the priorities outlined here with large comparative effectiveness research studies.

Patient engagement has the potential to enrich our understanding of patient priorities for research. Given the current focus on developing patient‐centred research questions, we suggest future studies to vigorously define and evaluate various patient engagement approaches and determine what approach works best, under which circumstances. Successful approaches will build trust in the patient‐research partnership, ensure that patients are meaningfully engaged throughout the process and capture the diversity of patient experiences and perspectives. The detailed patient engagement approach we used in this study provides a blueprint for groups interested in pursuing a collaborative approach in which multi‐stakeholder groups work together to identify research priorities.

## CONFLICT OF INTEREST

All authors declare that he/she has no conflict of interest of this study.

## AUTHORS’ CONTRIBUTION

AY, SU, AW and MS conceived and planned the current study. AY wrote the manuscript with input from all authors. CP, DN, KD and RM contributed to the implementations of the project. MS helped supervise the project. MJK, LE and MS provided critical revision of the article. All authors discussed the results and contributed to the final manuscript.

## Data Availability

The data used to support the findings of this study are available from the corresponding author upon request.
